# Effect of Solution Viscosity on the Precipitation of PSaMA in Aqueous Phase Separation-Based Membrane Formation

**DOI:** 10.3390/polym13111775

**Published:** 2021-05-28

**Authors:** Wouter M. Nielen, Joshua D. Willott, Julia A. R. Galicia, Wiebe M. de Vos

**Affiliations:** Membrane Surface Science (MSuS), Membrane Science and Technology Cluster, Mesa+ Institute for Nanotechnology, University of Twente, P.O. Box 217, 7500 AE Enschede, The Netherlands; w.m.nielen@utwente.nl (W.M.N.); j.d.willott@utwente.nl (J.D.W.); j.a.rodriguezgalicia@student.utwente.nl (J.A.R.G.)

**Keywords:** polyelectrolytes, membranes, sustainable, aqueous phase separation, contaminants of emerging concern

## Abstract

Aqueous phase separation (APS) is a recently developed sustainable alternative to the conventional organic solvent based nonsolvent-induced phase separation (NIPS) method to prepare polymeric membranes. In APS, polyelectrolytes are precipitated from aqueous solutions through pH or salinity switches. Although APS differs from NIPS in the polymer and solvents, they share many tuning parameters. In this work, we investigate the APS-based preparation of membranes from poly(styrene-alt-maleic acid) (PSaMA) with a focus on acid concentration in the coagulation bath, and polymer and additive concentration in the casting solution. Nanofiltration membranes are prepared using significantly lower concentrations of acid: 0.3 M HCl compared to the 2 M of either acetic or phosphoric acid used in previous works. It is shown that higher polymer concentrations can be used to prevent defect formation in the top layer. In addition, acetic acid concentration also strongly affects casting solution viscosity and thus can be used to control membrane structure, where lower acetic acid concentrations can prevent the formation of macrovoids in the support structure. The prepared nanofiltration membranes exhibit a very low molecular weight cutoff (210 ± 40 dalton), making these sustainable membranes very relevant for the removal of contaminants of emerging concern. Understanding how the parameters described here affect membrane preparation and performance is essential to optimizing membranes prepared with APS towards this important application.

## 1. Introduction

The preparation of polymeric membranes through nonsolvent-induced phase separation (NIPS) has been extensively investigated since its invention in the 1960s [1,2,3,4]. With NIPS, it is possible to prepare defect-free membranes in a simple and scalable way for a wide range of applications [3,5]. In addition, there are many system variables that can be used to control membrane structure. The typical tuning parameters for the phase separation include: choice of solvent and polymer [6], polymer concentration [5,7,8], additives in the polymer solution [9,10,11], composition of the nonsolvent [12,13,14], and temperature [15,16]. Through precise tuning of these parameters, the flux and retention of the resultant membranes can be optimized towards a specific application. In recent years there has been quite some research into preparing membranes using different variants of NIPS in an effort to find more sustainable alternatives [17,18]. Most of this research has focused on using nontoxic or green solvents instead of the commonly used N-methyl pyrrolidone (NMP) or dimethylformamide (DMF), which are reprotoxic and used in large quantities resulting in large amounts of contaminated waste water [18,19]. The alternative solvents include solvents like dimethyl sulfoxide (DMSO) [20], supercritical CO_2_ [21], ionic liquids [22], and new synthetic solvents [23,24].

An especially promising alternative to NIPS is APS, where water acts as both the solvent and the nonsolvent during membrane fabrication [25,26,27,28,29,30]. To facilitate this, polyelectrolytes, which are highly charged polymers, are used as the polymer/membrane material. Polyelectrolytes are divided into two categories, strong or weak, depending on the nature of the charged group. Weak polyelectrolytes, such as poly(acrylic acid) and poly(ethylene imine), are highly sensitive to pH and can be either charged or uncharged based on their environment. Strong polyelectrolytes, such as poly(styrene sulfonate) or poly(diallyldimethylammonium), are typically always charged and are generally insensitive to pH but are still sensitive to salt concentration. APS uses a switch in pH or salinity to precipitate polyelectrolytes from the solution to form membranes [25,26,27,28,29,30]. Although very different polymers and solvents are used, most tuning parameters used in NIPS can also apply to APS systems. For example, the polymer concentration is an important parameter commonly used in NIPS which can also be applied to the APS approach. Here, an increased polymer concentration leads to an increased solution viscosity through increased polymer entanglement, and vice versa. In NIPS, a low polymer concentration is used to prepare more open structures, while an increased concentration is used for denser structures with smaller pore sizes [2,7]. In the recent work of Baig et al. [29], where a pH shift is used to induce a polyelectrolyte-complexation-induced precipitation, it was observed that polymer concentration has a strong effect on the pore size and permeability, following the exact same trend as in regular NIPS. Additives to the polymer casting solution are another commonly used tuning parameter in NIPS, which has parallels in APS. In NIPS, the addition of nonsolvent molecules or polymers can be used to suppress macrovoid formation, tune the solution viscosity, and improve pore formation and interconnectivity [4]. In previous works on APS, similar results were observed, where the addition of acetic acid to the polymer casting solution strongly affected the solution viscosity and membrane structure [26,28]. Finally, nonsolvent composition is another strong tuning parameter in NIPS for which similar effects can be found in APS. In regular NIPS, the addition of solvent to the nonsolvent is commonly employed to lower the driving force of precipitation and prevent instant demixing, which can be used either to prepare denser membranes [6,31] or open membranes in combination with other parameters [32]. In the work of Durmaz et al., it was observed that while using a salinity driven APS method in which salt can be seen as the solvent, the addition of salt to the nonsolvent coagulation bath resulted in membranes with lower permeabilities and higher retentions. On the other hand, in APS methods that use a pH switch, it has been observed that a lower pH difference between the polymer solution and nonsolvent results in more open structures [26,33]. While the previously mentioned parameters appear to be quite similar for both NIPS and APS, there are also a new set of tuning parameters.

Due to the highly charged nature of polyelectrolytes, the system is sensitive to new parameters. We know from other works that salt type and concentration play an important role in the behavior of polyelectrolytes [34] and can strongly affect the formation and resulting properties of membranes [27,29,30,35]. It was found that when preparing membranes with poly(stryrene-alt-maleic acid) (PSaMA), the addition of LiCl to the coagulation bath led to more open structures, while CaCl_2_ densified the membrane [35]. In systems were two oppositely charged polyelectrolytes undergo complexation to form membranes, it has been found that the mixing ratio of the two polyelectrolytes is an additional parameter to control the phase separation [30,33,36]. This shows while there are many similarities, there are also intrinsic differences in the APS method compared to NIPS which need to be thoroughly investigated to fully comprehend the system.

In this work, the focus lies on further investigating the APS system where membranes are prepared using PSaMA. This method uses HCl and acetic acid instead of reprotoxic solvents to prepare membranes. In previous research, it was found that membranes were most easily prepared using 2 M of either acetic (12.0% *w*/*w*) or phosphoric acid (19.6% *w*/*w*) in the coagulation baths [26,35]. While the sustainability of acetic acid is not a problem, as it can be biosourced [37], waste streams with large amounts of acid are problematic, and therefore using less acid is better. Here, we aim to further improve the sustainability of this APS method by using much lower acid concentrations to precipitate PSaMA. In conjunction with this, the effect of viscosity of the polymer casting solution is investigated to determine how this can be used to improve membrane structure and performance. This is done either through an increased polymer concentration, which increases polymer entanglements, or a reduction of acetic acid in the casting solution, which increases inter-/intrapolymer hydrogen bonding. Through the tuning of these parameters, the porosity of the top layer as well as the overall membrane structure is controlled. The nanofiltration membranes prepared in this work have a low molecular weight cutoff, which allows them to effectively remove micropollutants. With these membranes it is possible, in a sustainable way, to remove these pollutants, which are currently appearing with increasing concentrations in waste waters, surface waters, and even drinking waters [38,39,40].

## 2. Experimental Section

### 2.1. Materials

Poly(styrene-alt-maleic acid) sodium salt solution 13% (M_w_ 350,000 g·mol^−1^, PSaMA), polyethylene glycol (M_w_ 200 g·mol^−1^, PEG 200; M_w_ 400 g·mol^−1^, PEG 400; M_w_ 600 g·mol^−1^, PEG 600; M_w_ 1500 g·mol^−1^, PEG 1500; M_w_ 2000 g·mol^−1^, PEG 2000), Dextran from Leuconostoc spp./mesenteroides (Mw 60,000–76,000 Mw 100,000–200,000, Mw 450,000–650,000), (polyethyleneimine, branched (M_n_ 600 g·mol^−1^, PEI 600), N-(3-Dimethylaminopropyl)-N′-ethylcarbodiimide hydrochloride (EDC), N-Hydroxysuccinimide (NHS), atenolol, atrazine, bezafibrate, bisphenol A, naproxen, sulphamethoxazole, magnesium sulfate, magnesium chloride, sodium sulfate, glacial acetic acid, and hydrochloric acid 37% were purchased from Sigma-Aldrich. Ethanol 100% technical grade was bought from Boom B.V. N-hexane 99+% was purchased from Acros organics. Sodium chloride (Sanal^®^ P) was received from AkzoNobel. Deionized water (DI, 1.0 µS·cm^−1^) was used for the preparation of coagulation baths and Milli-Q water (Millipore, 0.6 μS·cm^−1^) was used to prepare solutions. Apart from PSaMA, which was dried at 100 °C for 16 h, all chemicals were used as received.

### 2.2. Membrane Preparation

Membranes were prepared using the methods described in our previous works with the vital details reported below, where Table 1 gives an overview of all conditions used to prepared membranes in this study [26,35]. Polymer casting solutions were prepared by dissolving PSaMA in water mixed with acetic acid. After homogenous solutions were obtained through agitation on a roller bank, they were filtered through a Bekaert 25 µm Bekipor ST25 AL 3 steel filter. Solutions were typically allowed to rest for 24 h to degas. Membranes prepared in coagulation baths containing 0.1 M HCl or less were cast onto a nonwoven fabric (polyphenylene sulfide) for additional mechanical support, while membranes prepared at higher concentrations of HCl were cast onto a glass substrate. It is unlikely that the type of support has a significant effect on the selective layer of the membrane. Regardless of the substrate, a steel casting knife with a 0.3 mm gap height was used. After casting, the membranes were immediately submerged into the coagulation bath (± 20 L per m^2^ of membrane), typically for 5 min, although some membranes prepared in 0.1 M HCl coagulation bath were left for up to 15 min to ensure complete precipitation. After precipitation, the membranes were rinsed twice using 0.2 M HCl baths (±20 L per m^2^ of membrane). To improve chemical stability and prevent PSaMA from dissolving at a neutral pH, an aqueous carbodiimide based crosslinking mechanism with low molecular weight PEI as the crosslinker was used [41]. Crosslinking concentrations were based on the amount of carboxylic acid groups using Equation (1):

(1) $$C=\frac{h\cdot A\cdot w\cdot a}{M_{w}}$$

where C is the number of carboxylic acid groups, h the gap height of casting knife, A the surface area of the membrane, w the % *w*/*v* of polymer in the casting solution, a the amount of acid group per polymer chain, and $M_{w}$ the molecular weight of the polymer. Typically a 1:1:0.4:0.33 ratio of carboxylic acid groups: EDC:NHS:PEI was used. Using HCl, the pH of the crosslinking bath was set to approximately 5. After crosslinking, membranes were washed twice for 30 min using DI water.

### 2.3. Membrane Performance Tests

To assess membrane performance, stirred dead-end filtration cells with a pressurized feed vessel were used in a similar fashion as in our previous works [26,35]. Free standing membranes with 380 mm^2^ permeable surface area supported by nonwoven fabric were studied for pure water permeability and their ability to retain salts, PEG, dextran, or micropollutants at 4 bars of applied pressure. In addition, 5 mM salt solutions of the various salts were used, and the conductivity of the feed, retentate, and permeate was used to calculate the retention with Equation (2):

(2) $$R=\left(1-\frac{C_{p}}{\frac{C_{f}+C_{r}}{2}}\right)\cdot 100\%$$

where R is the retention, and *C_p_*, *C_f_*, and *C_r_* are the conductivity in the permeate, feed, and retentate, respectively.

The molecular weight cutoff measurements were performed using 1 g·L^−1^ of the various PEG or dextran molecules; in case of dextran, 0.1 g·L^−1^ ethylene glycol was used as an internal standard. Samples were analyzed via gel permeation chromatography (Agilent 1200/1260 Infinity GPC/SEC series, Polymer Standards Service data center and column compartment). For PEG measurements, Milli-Q eluent containing 50 mg·L^−1^ NaN_3_, at 1 mL·min^−1^, and two Polymer Standards Service Suprema 8 × 300 mm^2^ columns in series: 1000 Å, 10 µm, followed by 30 Å, 10 µm were used. For dextran measurements, Milli-Q eluent containing 50 mg·L^−1^ NaN_3_ and 0.1 M NaNO_3_, at 1 mL·min^−1^, and four Polymer Standards Service Suprema 8 × 300 mm^2^ columns in series: Guard 5 µm, 1000 Å, 5 µm, 1000 Å, 5 µm, and 30 Å, 5 µm were used. Concentrations were calculated via refractive index detection, and with Equation (2), the retention for the different molecules was calculated, where R is the retention, and *C_p_*, *C_f_*, and *C_r_* are the concentration in the permeate, feed, and retentate, respectively.

The micropollutant retention was measured at 4 bars of applied pressure with a 3 mg·L^−1^ solution of atenolol, atrazine, bezafibrate, bisphenol A, naproxen, and sulfamethoxazole set to pH 5.8. To account for the absorption permeate, samples were taken after 24 h of continuous permeation, at which point a steady state between absorption and desorption was assumed [42]. The concentration of the micropollutants in the sample were determined using ultra-high performance liquid chromatography (Dionex Ultimate 3000, water/acetonitrile gradient, 0.1% phosphoric acid, 0.8 mL·min^−1^) through a Thermo Scientific Acclaim RSLC 120 C18 column (2.2 µm, 2.1 × 100 mm^2^), with UV/Vis detection at 225 nm. Using Equation (2), the retention was calculated.

### 2.4. Scanning Electron Microscopy (SEM)

SEM samples were prepared using the same method as in previous works. A solvent exchange with ethanol (twice for 30 min) followed by hexane (twice for 30 min) was used to remove water and prevent pore collapse during drying. Using liquid nitrogen, the samples were fractured and mounted on sample holders. After at least 4 h in a vacuum oven at 30 °C the samples were coated with 5 nm platinum–palladium using a Quorum Q150T ES and imaged using a Jeol JSM-6010LA scanning electron microscope.

### 2.5. Dynamic Viscosity Measurements

The dynamic viscosity of the polymer solutions was measured using a HAAKE Viscotester 550 Rotational Viscometer with a SV-DIN rotor and cup, with shear rates 2.6–258 s^−1^ and temperature 20–60 °C.

## 3. Results and Discussion

This work investigates how acid concentration in the coagulation bath and polymer casting solution viscosity affect the preparation of membranes using PSaMA in an aqueous phase separation system. To further improve the sustainability of the method, a significantly reduced acid concentration in the coagulation bath is used compared to previous works [26,35]. The effect of the polymer casting solution viscosity is investigated by systematically changing the polymer and acetic acid concentrations in the casting solution.

### 3.1. Reduced Acid Concentration

**Figure 1:**
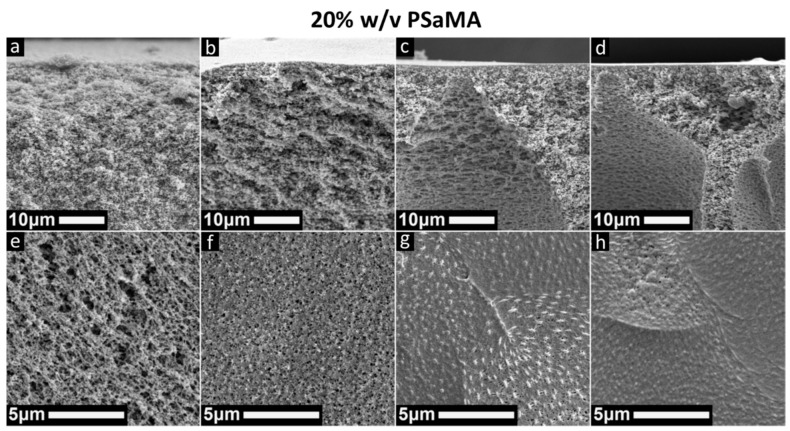
SEM images of cross sections and top surfaces of membranes prepared in a coagulation bath with 0.05 M HCl (**a**,**e**), 0.1 M HCl (**b**,**f**), 0.2 M HCl (**c**,**g**), and 0.3 M HCl (**d**,**h**), using a 20% *w*/*v* PSaMA, 40% *v*/*v* acetic acid polymer casting solution.

In previous works, membranes prepared with PSaMA were typically precipitated in coagulation baths consisting of either 2 M acetic or phosphoric acid [26,35]. Such large amounts of weak acid were used as it was shown that hydrogen bonding between the polymer weak acid played a significant role during precipitation. In this work, to further improve the sustainability of this APS method, the use of lower acid concentrations using HCl instead of acetic acid was investigated. As HCl is a strong acid, lower concentrations are required to reach the pH at which PSaMA precipitates; in addition, it lacks the buffer capacity that weak acids have, which can potentially lead to different membrane morphologies. Using the same polymer casting solution as before (20% *w*/*v* PSaMA with 40% *v*/*v* acetic acid), membranes were prepared in coagulation baths containing 0.05–0.3 M HCl, of which the SEM images can be seen in Figure 1. A general trend is observed where an increasing acid concentration results in membranes with denser top layers, which is as expected, as the acid concentration is the main driving force for the precipitation of PSaMA [26]. Membranes prepared in 0.05 M HCl have a very open top layer, and the entire film has poor mechanical properties. It has been observed previously that membranes with structures like this have a very low water permeability as the top layer collapses on itself due to the membrane’s poor mechanical stability [35]. The resulting dense support structure is expected to have a high resistance to water. This behavior was confirmed, in that the pure water permeability of the membrane was only 23 ± 1 L·m^−2^·h^−1^·bar^−1^, two orders of magnitude lower than comparable porous membranes prepared in previous works [26,35]. The low permeability combined with the open selective layer as seen in the SEM images makes it unlikely that these membranes have any relevant selective properties and thus were not further investigated. The pure water permeability of the other membranes shown in Figure 1 is discussed later in the section on the effect of polymer concentration.

With 0.1 M HCl in the coagulation bath, a porous sponge-like structure is observed with a similar support structure to the one prepared with 0.05 M, but here the top layer appears to have a lower surface roughness. The structures prepared with 0.2 M and 0.3 M HCl in the coagulation bath are similar to each other, with macrovoids in the support structure and visible defects in the top layers. While most of the top surface is dense, at somewhat regular intervals, there are patches where pores are visible. It is hypothesized that these porous patches are formed due to shrinkage of the top layer. In some extreme cases, as seen in Figure S1, extensive patterning is observed, which is similar to patterns created when layers shrink as they dry [43,44]. It is possible that the patterns are formed during the sample preparation; however, as SEM samples were prepared with the same method as previous works where this effect was not observed, this scenario is unlikely. The porous nature of these surface patches is further confirmed by dextran separation experiments performed on membranes that were never dried. In Figure S2, the results of molecular weight cutoff measurements are shown for the membranes prepared in 0.2 M HCl. Figure S2 shows that the retention of dextran is almost completely independent of dextran size, indicating that these membranes contain a significant amount of so-called pinhole defects. Therefore, it can be assumed that the deformations of the top layer are not simply the result of drying. It is expected that at some point in the phase inversion or the crosslinking step, the top layer shrinks, causing the formation of the defects.

When comparing the coagulation bath conditions used to prepare the membranes in Figure 1 to those used in previous works, we observe that while pH is an important driving force for precipitation, it is only one a part of a complex system [26]. For instance, membranes prepared in 2 M formic acid, which is approximately pH 1.7, were asymmetric with a dense top layer [26], while Figure 1 shows that membranes prepared in pH 1 are symmetric and porous. An important difference between these two systems is the strong buffering capacity that weak acids have, especially at concentrations like 2 M. Calculations based on Equation (1) for required crosslinking concentrations show that a typical membrane with a 100 cm^2^ surface area has approximately 5.5 mmol acid carboxylic acid groups. At 0.1 M HCl there is only ±10 mmol HCl within 1 cm from the interface; although HCl has a high diffusion rate, the HCl concentration at the precipitation interface can be expected to drop as PSaMA is protonated. With a high concentration of a weak acid in the coagulation bath, the pH might be higher and the initial H^+^ concentration near the interface lower, but due to the buffer capacity, it remains more constant. Further increases in acid concentration of the coagulation bath did not yield better results, as seen in Figure S3 where similar structures as with 0.2 M and 0.3 M HCl are observed. It therefore appears that the defect formation in the selective layer is intrinsic to the conditions in which these membranes are prepared, and further investigation focused on changing the polymer casting solution instead of tuning the HCl concentration.

### 3.2. Polymer Concentration

**Figure 2:**
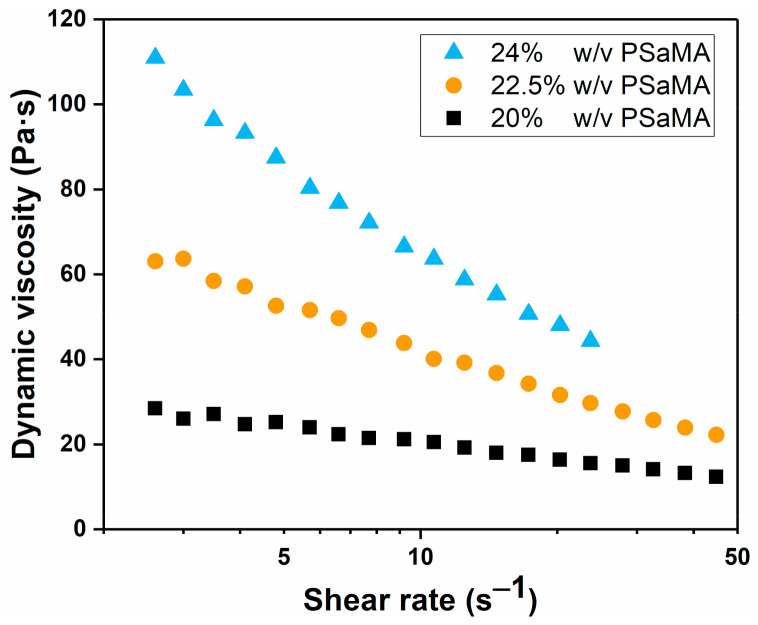
Dynamic viscosity for PSaMA solutions with 40% *v*/*v* acetic acid at different polymer concentrations, for different shear rates at 20 °C. The dynamic viscosity of the solution prepared with 24% *w*/*v* PSaMA reached the measurement limit of the viscometer at shear rates higher than 25 s^−1^. Data is from a single measurement.

It is well established that polymer concentration has a strong effect on the membrane formation, where increased polymer concentrations typically lead to denser structures and can suppress macrovoids [7]. An increased polymer concentration leads to more entanglements between polymers and therefore is expected to strengthen the top layer of the membranes and prevent it from shrinking, as was observed in Figure 1. Solutions were prepared at different concentrations of PSaMA while keeping the acetic acid concentration constant at 40% *v*/*v*. The dynamic viscosity measurements in Figure 2 show that as expected, the viscosity increases significantly at higher polymer concentrations, indicating increased entanglement of the polymers.

**Figure 3:**
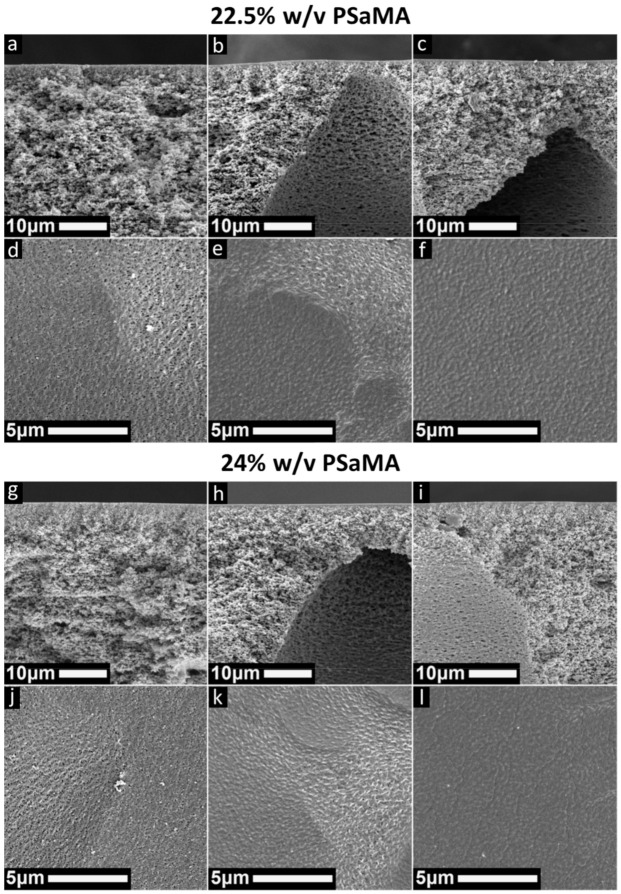
SEM images of cross sections and top surfaces of membranes prepared either with a 22.5% *w*/*v* PSaMA, 40% *v*/*v* acetic acid solution (top) or a 24% *w*/*v* PSaMA, 40% *v*/*v* acetic acid solution (bottom). Coagulation bath conditions are 0.1 M HCl (**a**,**d**,**g**,**j**), 0.2 M HCl (**b**,**e**,**h**,**k**), and 0.3 M HCl (**c**,**f**,**I**,**l**).

As with the 20% *w*/*v* PSaMA casting solutions discussed earlier, membranes were prepared with casting solutions with higher (22.5% and 24%) polymer concentration. As seen in Figure 3, when 22.5% *w*/*v* PSaMA is used, a similar trend in the morphology of the cross section is observed as when a 20% *w*/*v* PSaMA solution was used (Figure 1). At 0.1 M HCl, a symmetrical sponge-like structure is observed, while at higher acid concentrations (0.2, 0.3 M), an asymmetric structure with macrovoids is seen. When looking at the top surfaces, as before, similar patches with an increased porosity are observed. However, when 0.3 M HCl is used, these patches are not observed, indicating that the top layer of the membrane could now be defect free as desired. The structures of membranes prepared with 24% *w*/*v* PSaMA in the solution look very similar to those prepared with 22.5% *w*/*v* PSaMA. This indicates that the increased polymer concentration can indeed help prevent the shrinkage observed earlier in Figure 1; yet, a certain amount of driving force, in this case at least 0.3 M HCl, is required.

**Figure 4:**
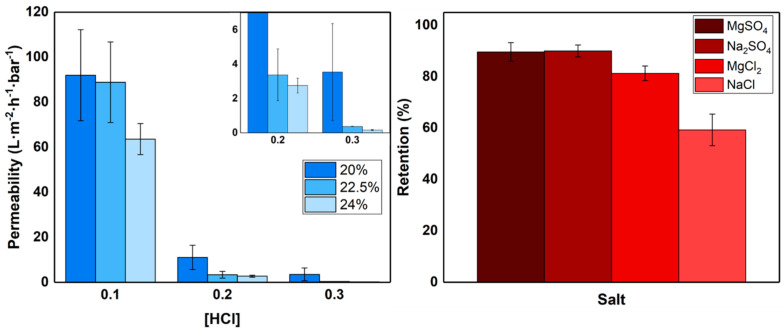
**Left**: pure water permeability of membranes prepared in a coagulation bath containing 0.1–0.3 M HCl using casting solutions containing either 20, 22.5, or 24% *w*/*v* PSaMA and 40% *v*/*v* acetic acid. The inset zooms in on the permeability of the membranes prepared in 0.2 M and 0.3 M HCl. **Right**: retention of various salts by membranes prepared with a 24% *w*/*v* PSaMA 40% *v*/*v* acetic acid solution in a coagulation bath with 0.3 M HCl. The error bars represent the sample standard deviation of at least three separate membranes.

To investigate the properties of the membranes prepared with the different polymer concentrations, their pure water permeabilities were measured (see Figure 4). As expected, based on the SEM images (Figure 1 and Figure 3), the acid concentration in the coagulation bath has the largest effect on permeability. A higher acid concentration leads to a denser top layer and thus a lower water permeability. For membranes prepared in the 0.1 M HCl coagulation bath, the influence of polymer concentration in the casting solution on the resultant membrane water permeability is relatively small. This is supported by their SEM images, as all structures look quite similar.

The membranes prepared in 0.2 M HCl have low water permeabilities, but as seen in the SEM images of Figure 1 and Figure 3, these membranes all have mostly dense top layers, albeit with a significant number of defects. From this, it is expected that most of the permeate flows through the defects, making them unsuitable for all membrane applications. To confirm this, filtration experiments were performed using dextran macromolecules of varying sizes. These dextran retention experiments showed, in Figures S2, S4, and S5, that retention is independent of molecular weight/size, proving that defects play a significant role during filtration.

**Figure 5:**
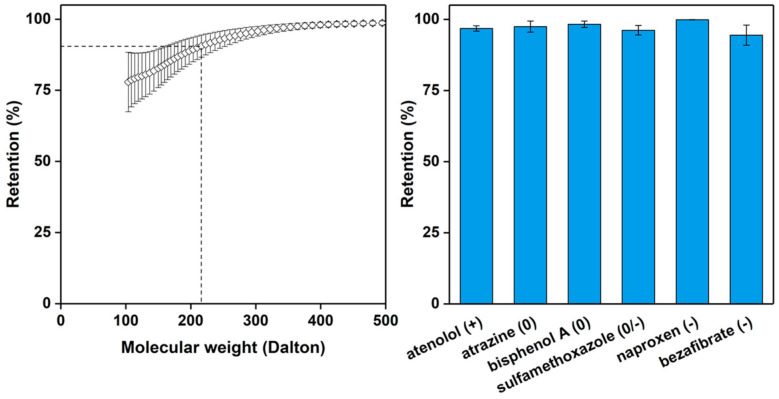
**Left**: Molecular weight cutoff; **Right**: retention of various micropollutants, of membranes prepared with a 24% PSaMA solution with 40% acetic acid in a coagulation bath with 0.3 M HCl. The 90% molecular weight cutoff is at 210 ± 40. The error bars represent the sample standard deviation of at least three separate membranes.

When 0.3 M HCl is used in the coagulation bath, the lowest water permeabilities are measured, especially for the membranes prepared from the 22.5% and 24% *w*/*v* PSaMA casting solutions. The low water permeability of these membranes is in agreement with the SEM images observed in Figure 3, indicating that the top layers are most likely defect free. The membrane prepared in 0.3 M HCl with the 24% *w*/*v* PsaMA solution was further investigated. The retention of various salts is shown in the right plot of Figure 4 and shows a relatively high overall retention with higher retentions for divalent salts compared to monovalent salts, similar to what was observed in previous works. This falls within expectations, as divalent ions such as Mg^2+^ and SO_4_^2^^−^ have large hydrodynamic radii and are therefore retained more than small monovalent ions such as Na^+^ and Cl^−^. The difference between Na_2_SO_4_ and MgCl_2_ can be explained by electrostatic interaction with a large amount of the negatively charged carboxylate groups in the membrane, which makes it easier for large positively charged ions to permeate. To investigate the retention of uncharged molecules, molecular weight cutoff measurements were performed using PEG, as seen in Figure 5 on the left. The low molecular weight cutoff of 210 ± 40 further demonstrates that these membranes are dense nanofiltration membranes. Further performance analysis of these membranes was done using a mixture of multiple small organic micropollutants (216–361 dalton) with various charged states. The membranes show a very high retention for all these micropollutants (97 ± 1.9%), demonstrating their relevance to removing emerging contaminates in waste waters, surface waters, and drinking waters, even though the water permeability of these membranes is low [38,39,40]. This shows that polymer concentration, as in classical NIPS, is a very important parameter that can be used to tune the APS process, enabling the formation of defect-free dense NF membranes with very high retentions at bath acid concentrations as low as 0.3 M HCl. The performance of these membranes is very similar to those prepared in previous work [26,35] with 2 M acetic (12.0% *w*/*w*) or phosphoric acid (19.6% *w*/*w*), demonstrating that such acid concentrations are not required to obtain NF membranes with high retentions.

### 3.3. Acetic Acid Concentration

**Figure 6:**
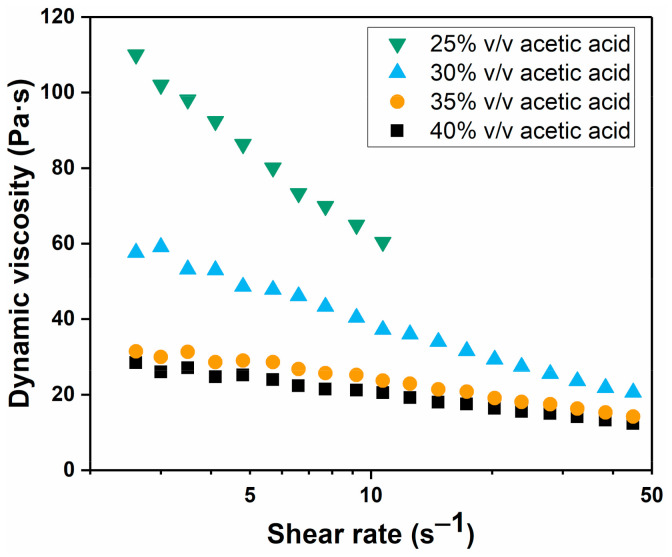
Dynamic viscosity for 20% *w*/*v* PSaMA solutions with different concentrations of acetic acid for different shear rates at 20 °C. The dynamic viscosity of the solution prepared with 20% *w*/*v* PSaMA 25% *v*/*v* acetic acid reached the measurement limit of the viscometer at shear rates higher than 10 s^−1^. Data is from a single measurement.

As shown in this work and in the literature, polymer concentration has a strong effect on the phase separation behavior [7,8]. Polymer concentration directly affects solution viscosity, which in turn influences the diffusion of different species during the phase separation process. Besides polymer concentration, additives can also be used to control solution viscosity. In this APS system, as in our previous work, acetic acid is one such additive whose concentration strongly affects the viscosity of the polymer casting solution. Figure 6 shows the dynamic viscosity of solutions prepared with different concentrations of acetic acid. Here, an increase in the acetic acid concentration results in a decreased viscosity until a certain minimum is reached, which in this case appears to be around 40% *v*/*v* [26]. Lowering the acetic acid concentration results in a higher viscosity, and with less than 25% *v*/*v* acetic acid, it becomes increasingly difficult to obtain a solution.

The behavior of acetic acid in this system is complex, as acetic acid affects both the kinetics of the phase inversion, by its effect on the viscosity, and the thermodynamics, as it is a nonsolvent for PSaMA, but its strength as a nonsolvent strongly depends on the concentration. At low concentrations, it results in the precipitation of PSaMA, but at higher concentrations, as seen in Figure 6, the solution viscosity is inversely dependent on the acetic acid concentration. The reason acetic acid affects the solution viscosity in this manner is that it lowers the solution pH and therefore changes the polymer by partially protonating the acid groups. The protonated carboxylic acid groups of PSaMA can form hydrogen bonding pairs with other carboxylic acid groups which causes an increase in inter-/intrapolymer interaction, resulting in an increased viscosity. However, since acetic acid is a weak acid, at the concentrations used here, ±99% of it is in the protonated form in which it can also form hydrogen bonding pairs with PSaMA. Therefore, at a high concentration, e.g., 40% *v*/*v*, acetic acid behaves like a solvent, significantly reducing the inter-/intrapolymer hydrogen bonding interactions of PSaMA and thus lowering the solution viscosity. At lower concentrations, such as 25% *v*/*v* acetic acid, the effect is less pronounced and an increase in inter-/intrapolymer interactions resulting in an increased viscosity is observed; thus, acetic acid acts similar to a nonsolvent. When comparing the viscosity data in Figure 6 to the viscosity data from our previous work [26], it is observed that the viscosity of the solutions used in this work is lower than of those used in the previous work, most likely due to differences in the batch of polymer used. It is also observed that the viscosity of solutions with low acetic acid concentrations is strongly temperature dependent due to the dynamic nature of hydrogen bonds. The viscosities of the 20% *w*/*v* PSaMA 25% *v*/*v* acetic acid solution and the 24% *w*/*v* PSaMA 40% *v*/*v* acetic acid solution are quite similar at room temperature, but, as seen in Figure S6, at elevated temperatures, the viscosity of the 20% *w*/*v* PSaMA 25% *v*/*v* acetic acid solution is significantly lower than that of the 24% *w*/*v* PSaMA 40% *v*/*v* acetic acid solution. While this is an interesting phenomenon, due to the impracticality of preparing flat sheet membranes at elevated temperatures, this was not further investigated.

**Figure 7:**
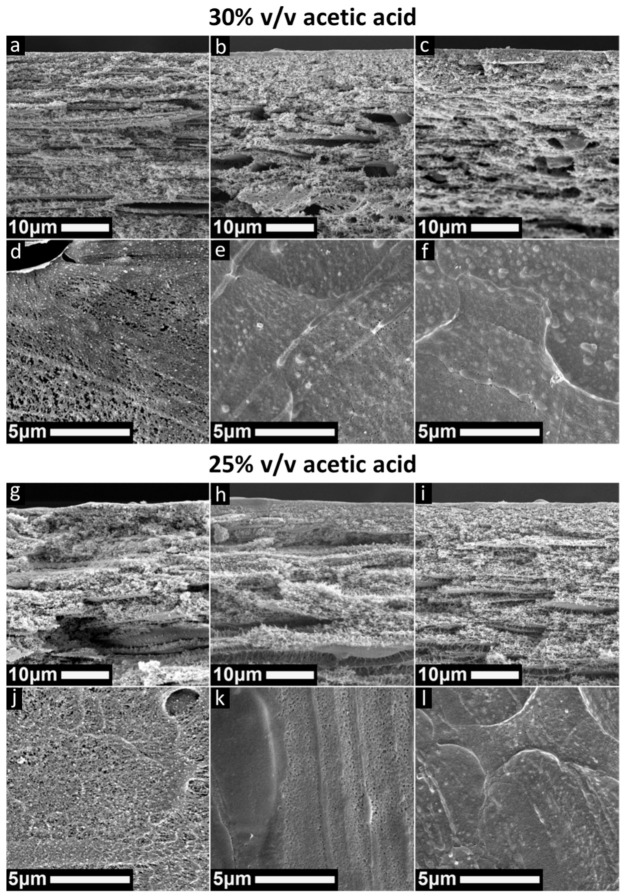
SEM images of cross sections and top surfaces of membranes prepared either with a 20% *w*/*v* PSaMA, 30% *v*/*v* acetic acid solution (top) or a 20% *w*/*v* PSaMA, 25% *v*/*v* acetic acid solution (bottom). Coagulation bath conditions are 0.1 M HCl (**a**,**d**,**g**,**j**), 0.2 M HCl (**b**,**e**,**h**,**k**), and 0.3 M HCl (**c**,**f**,**i**,**l**).

In Figure 7, SEM images of membranes prepared with casting solutions of 20% *w*/*v* PSaMA and 30% *v*/*v* acetic acid and 20% *w*/*v* PsaMA and 25% *v*/*v* are shown. The most striking difference is that regardless of the acid concentration used in the coagulation bath, no macrovoids are observed in the cross-section images. Secondly, the top layers of the membranes prepared in 0.1 M HCl are quite irregular and at higher acid concentrations in the coagulation bath they are similar to before, with a mostly dense top layer with occasional defects. The large differences in the support structure of the membranes prepared with 0.2 M and 0.3 M HCl are unlikely to be solely caused by the effect of viscosity, as it would also have been observed earlier when an increased polymer concentration was used. Most likely the differences in support structure are caused by how the reduced acetic acid concentration changes the behavior of the polymer casting solution.

Initial expectations were that a high concentration of acetic acid present in the film during precipitation acts as solvent and delays the precipitation. Due to the dense layer that quickly forms on contact with the coagulation bath, the diffusion of acetic acid out of the film is impeded, and this prolonged high concentration of acetic acid in the support structure slows precipitation, allowing macrovoids to form. Following that logic, it would be safe to assume that by starting with a lower acetic acid concentration in the polymer casting solution the precipitation of the support is more rapid, and this suppresses the formation of macrovoids. This behavior appears to be confirmed by the SEM images in Figure 7 where no macrovoids are observed.

Yet, when the speed of precipitation of all membranes prepared in this study was analyzed, as seen in Figures S7 and S8, it was observed that a lower acetic acid concentration in the polymer casting solution significantly slows down the total speed of precipitation. The SEM images show that the top layers are quite comparable regardless of which polymer casting solution is used. This indicates that the morphology of the top layer is mostly determined by the acid concentration in the coagulation bath and that acetic acid concentration in the polymer casting solution has the biggest effect on the supporting structure. It is expected that these differences are largely caused by the inter-/intra hydrogen bonding of PSaMA and its interactions with PSaMA.

While the difference in dynamic viscosity of the 24% *w*/*v* PSaMA 40% *v*/*v* acetic acid and 20% *w*/*v* PSaMA 25% *v*/*v* acetic acid polymer casting solutions is small, the origin of their viscosity is significantly different. The high viscosity of the solution with 24% *w*/*v* is caused by an increased amount of polymer entanglement compared to a 20% *w*/*v* solution. For the solution with only 25% *v*/*v* acetic acid, the increased viscosity is caused by a strong increase in inter-/intrapolymer hydrogen bonding, which is significantly more dynamic than polymer entanglements. This is observed by the increased response to temperature, as seen in Figure S6, and also a slight elastic behavior of the solution. This elasticity is most likely caused by the inter-/intrapolymer hydrogen bonding, as this allows the formation of large dynamic supramolecular structures that can be broken under shear stress, which can be compared to shear thinning hydrogen-bond-based hydrogels [45]. Further indication of this can be found in the cross-section SEM images that show a somewhat laminar structure in which occasional fiber like structures are observed (see Figure S10). It is hypothesized that these structures are formed by the shear stress caused by the casting of the film where the hydrogen bonded supramolecular formations are stretched and broken apart. With the high viscosity and the formation of new hydrogen bonds, these structures do not have time to relax before precipitation occurs and can therefore be observed. Due to the immobile nature of these large supramolecular complexes, the kinetics of the phase separation are significantly slowed down and macrovoids cannot form, resulting in the spongy structure observed in the SEM images, even though the precipitation kinetics are extremely slow. Therefore, our hypothesis is that the difference in the precipitation speed of the solutions with lower acetic acid concentrations (25–30% *v*/*v*) compared to those prepared with 40% *v*/*v* acetic acid is also caused by these large supramolecular complexes, which create a gel like phase that strongly slows down the kinetics during the phase separation process, severely hindering the exchange of acid and solvents. Further research will be needed to confirm this, and unfortunately, as seen in the SEM images and the relatively high permeability (Figure S11), the selective layers of these membrane still have multiple defects. Nonetheless, this research provides interesting and relevant information for further optimizing this APS system, as it demonstrates a method to prepare macrovoid-free membranes, which is of interest when membranes with high mechanical stability are desired.

## 4. Conclusions

Using the APS approach, membranes were prepared with PSaMA at reduced acid concentrations to further improve the sustainability of the method. Using 0.1 M HCl in the coagulation bath, open, porous membranes could be prepared, albeit with a low water permeability. At higher acid concentrations, dense membranes were prepared, but these contained multiple defects in the top layer, making them unsuitable for separation purposes. The problem of defect formation was overcome by increasing the polymer concentration in the casting solution (24% *w*/*v* instead of 20% *w*/*v* PSaMA), with which defect-free dense membranes were prepared in acid concentrations as low as 0.3 M HCl. These membranes showed high salt retentions as well as a low molecular weight cutoff (210 ± 40) well within the nanofiltration regime. Furthermore, very high retentions (on average 97 ± 1.9%) for various small organic micropollutants (216–361 dalton) were obtained, which demonstrates the relevance of these membranes for the removal of emerging contaminants. A recurring issue, however, is that the water permeability of NF membranes prepared with PSaMA is, as in previous works, quite low. In previous work [26,35], it was shown that it is possible to prepare open membranes with a high-water permeability. Therefore it would be interesting to focus future investigation for preparation of NF membranes on using PSaMA membranes as a support for composite membranes.

Besides polymer concentration, the acetic acid concentration (an additive) in the polymer casting solution has also shown to be an important control parameter in the phase separation process. Acetic acid regulates the extent of inter-/intrapolymer hydrogen bonding between PSaMA in the casting solution, where the acetic acid concentration is inversely correlated to the solution viscosity. It is observed that at 25–30% *v*/*v* acetic acid, the kinetics of the phase separation are significantly slower compared to solutions with a higher acetic acid concentration. The slowed kinetics are most likely caused by the formation of large supramolecular complexes formed by hydrogen bonding and can be used to suppress the formation of macrovoids in dense asymmetric membranes. Increasing the polymer concentration and lowering the acetic acid concentration both result in an increased viscosity. However, interestingly the polymer concentration mostly seems to affect the structure of the selective layer, while the acetic acid concentration mostly affects the morphology of the support structure. Although no defect-free membranes were prepared using a reduced acetic acid concentration, a greater understanding of the precipitation behavior of PSaMA and its interaction with acetic acid is obtained. As the effects of a low acetic acid concentration on viscosity of the polymer casting solution are strongly temperature dependent, it is expected that temperature is also a very interesting parameter when membranes are prepared with PSaMA.

## Figures and Tables

**Table 1:** Overview of the conditions used to prepare membranes.

Polymer Solution Composition		Coagulation Bath Conditions [HCl]
PSaMA% *w*/*v*	Acetic Acid% *v*/*v*	
20	40	0.05, 0.1, 0.2, 0.3, 0.4, 0.5
20	30	0.1, 0.2, 0.1
20	25	0.1, 0.2, 0.1
22.5	40	0.1, 0.2, 0.1
24	40	0.1, 0.2, 0.1

## Data Availability

The data presented in this study are available in this article and its supplementary information.

## Supplementary Materials

The following are available online at https://www.mdpi.com/article/10.3390/polym13111775/s1, Figure S1. SEM image of the top surface of a membrane prepared with a PSaMA 20% *w*/*v* acetic acid 40% *v*/*v* solution in a coagulation bath with 0.2 M HCl. Figure S2. Molecular weight cut-off graph of membranes prepared with a 20% PSaMA solution with 40% acetic acid in a coagulation bath with 0.2M HCl. Figure S3. SEM images of cross sections and top surfaces of membranes prepared in a coagulation bath with 0.4 M HCl (a,c), 0.5 M HCl (b,d), using a 20% *w*/*v* PSaMA, 40% *v*/*v* acetic acid polymer casting solution. Figure S4. Molecular weight cut-off graph of membranes prepared with a 22.5% PSaMA solution with 40% acetic acid in a coagulation bath with 0.2M HCl. Figure S5. Molecular weight-cut off graph of membranes prepared with a 24% PSaMA solution with 40% acetic acid in a coagulation bath with 0.2M HCl. Figure S6. Dynamic viscosity measurements of a 24% *w*/*v* PSaMA 40% *v*/*v* acetic acid solution (left) and a 20% *w*/*v* PSaMA 25% *v*/*v* acetic acid solution for different shear rates at different temperatures (oC). The dynamic viscosity of the solutions at 20 oC reached the measurement limit of the viscometer. Data was taken from a single measurement. Figure S7. Frames taken at different times from movies of the precipitation of different polymer solutions in a 0.1 M HCl coagulation bath. Figure S8. Frames taken at different times from movies of the precipitation of different polymer solutions in a 0.2 M HCl coagulation bath. Figure S9. Frames taken at different times from movies of the precipitation of different polymer solutions in a 0.3 M HCl coagulation bath. Figure S10. SEM image of the cross section of a membrane prepared with a PSaMA 20% *w*/*v* acetic acid 30% *v*/*v* solution in a coagulation bath with 0.1 M HCl. Figure S11. Pure water permeability of membranes prepared in a coagulation bath containing 0.1–0.3 M HCl using casting solutions containing 20% *w*/*v* PSaMA and either 40%, 30% or 25% *v*/*v* acetic acid. The error bars represent the sample standard deviation of at least three separate membranes.
