# Supplementary material for: Effect of Solution Viscosity on the Precipitation of PSaMA in Aqueous Phase Separation-Based Membrane Formation
Source: Polymers (Basel). 2021 May 28;13(11):1775. doi: 10.3390/polym13111775 (PMC8198088; doi:10.3390/polym13111775)
Supplement: Supplementary file 1 [file polymers-13-01775-s001.zip › polymers-1214513-supplementary.pdf]

Effect of solution viscosity on precipitation of PSaMA in Aqueous Phase Separation based membrane formation.

Wouter M. Nielen,[1] Joshua D. Willott,[1] Julia A. Rodriguez Galicia ,[1] Wiebe M. de Vos[1]

[1] Membrane Surface Science (MSuS), Membrane Science and Technology cluster, Mesa+ Institute for Nanotechnology, University of Twente, P.O. Box 217, 7500 AE Enschede, the Netherlands

*corresponding author:

Keywords: polyelectrolytes, membranes, sustainable, water-based, aqueous phase separation

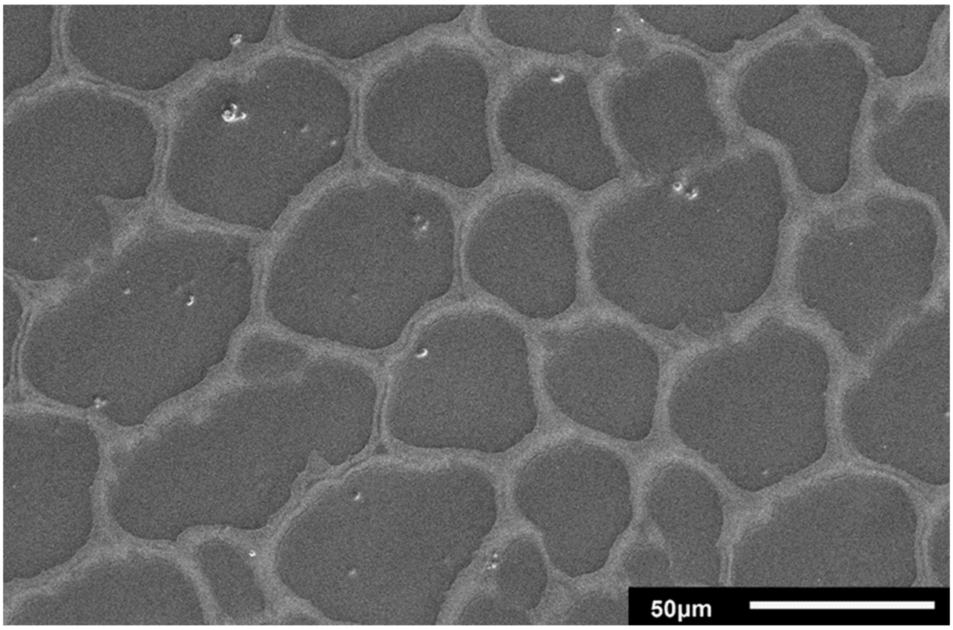

Figure S1. SEM image of the top surface of a membrane prepared with a PSaMA 20% w/v acetic acid 40% v/v solution in a coagulation bath with 0.2 M HCl.

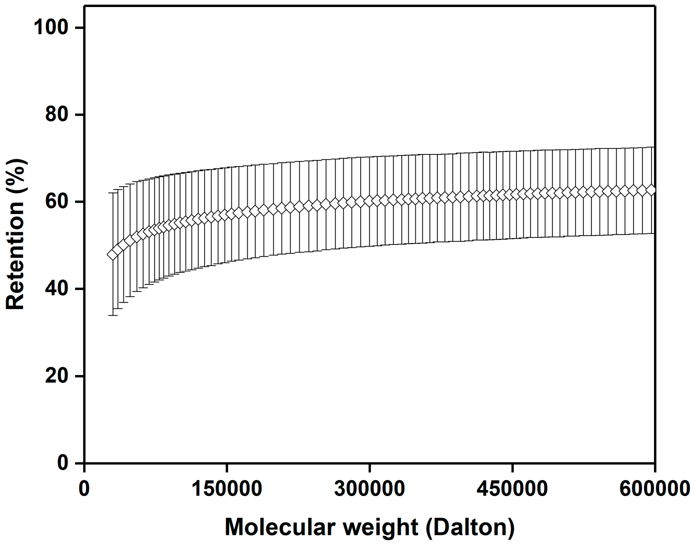

Figure S2. Molecular weight cut-off graph of membranes prepared with a 20% PSaMA solution with 40% acetic acid in a coagulation bath with 0.2M HCl.

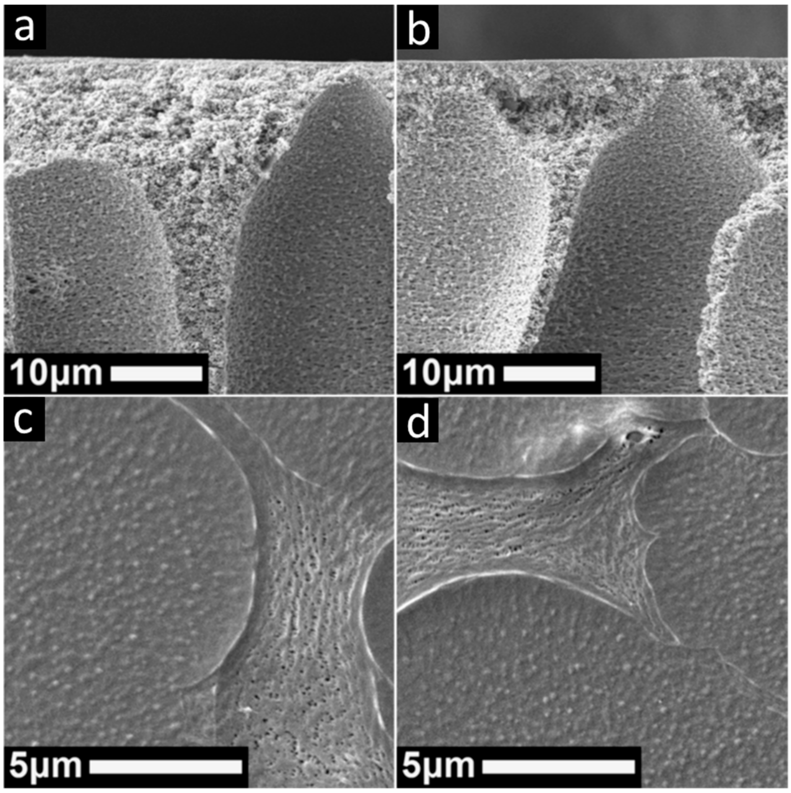

Figure S3. SEM images of cross sections and top surfaces of membranes prepared in a coagulation bath with 0.4 M HCl (a, c), 0.5 M HCl (b, d), using a 20% w/v PSaMA, 40% v/v acetic acid polymer casting solution.

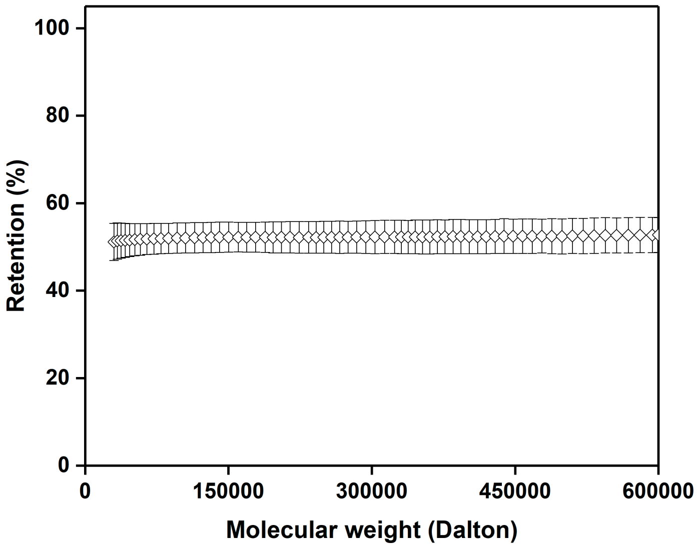

Figure S4. Molecular weight cut-off graph of membranes prepared with a 22.5% PSaMA solution with 40% acetic acid in a coagulation bath with 0.2M HCl.

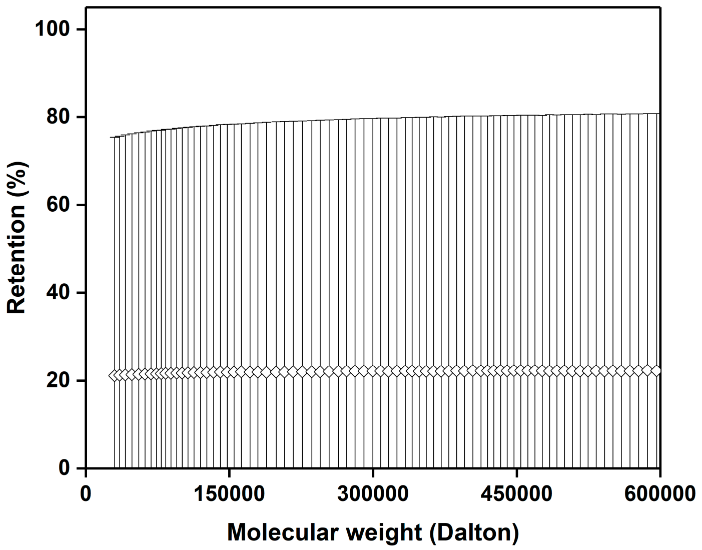

Figure S5. Molecular weight-cut off graph of membranes prepared with a 24% PSaMA solution with 40% acetic acid in a coagulation bath with 0.2M HCl.

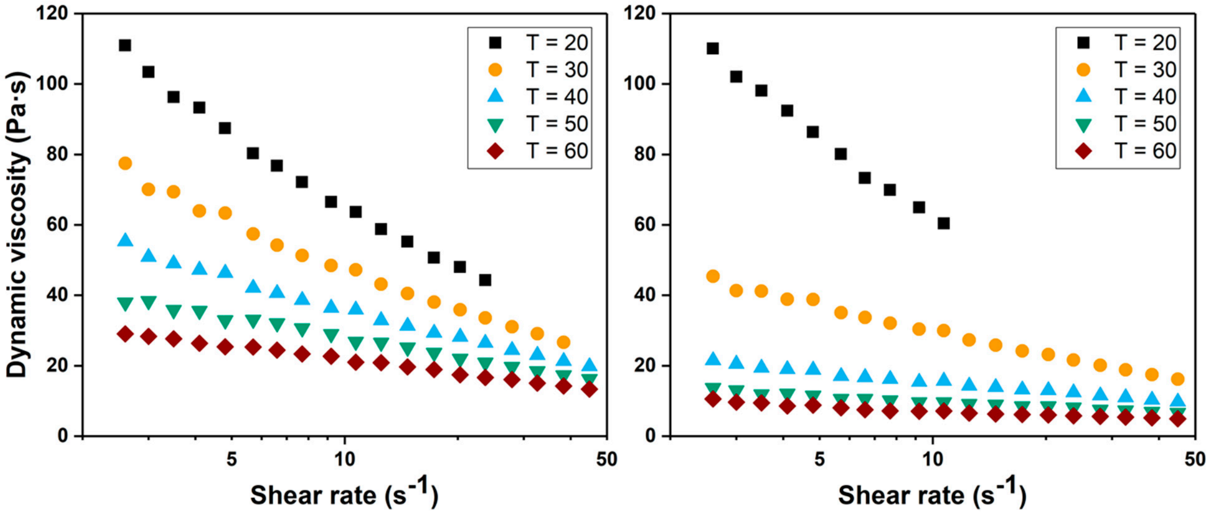

Figure S6. Dynamic viscosity measurements of a 24% w/v PSaMA 40% v/v acetic acid solution (left) and a 20% w/v PSaMA 25% v/v acetic acid solution for different shear rates at different temperatures (°C). The dynamic viscosity of the solutions at 20 °C reached the measurement limit of the viscometer. Data was taken from a single measurement.

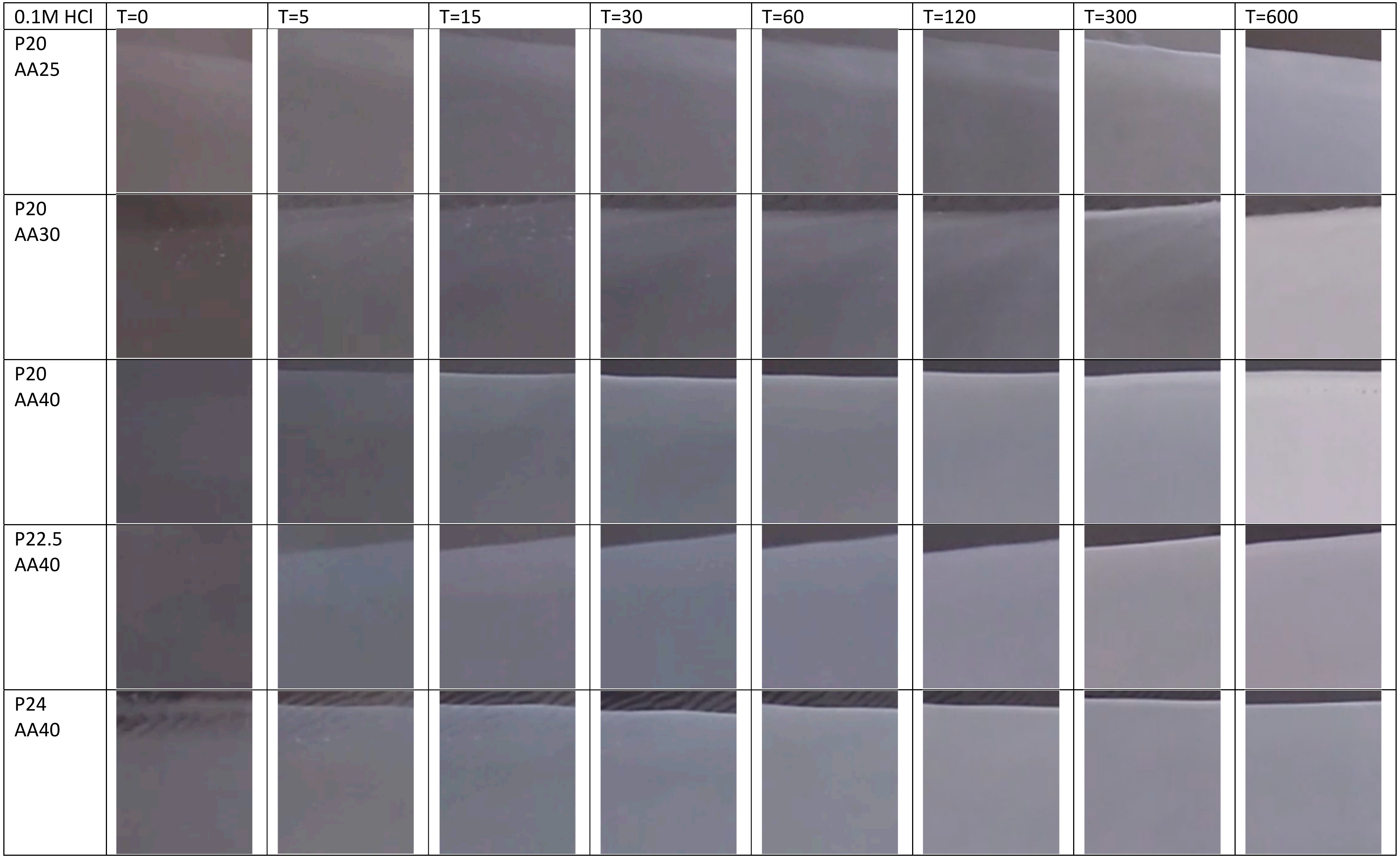

Figure S7. Frames taken at different times from movies of the precipitation of different polymer solutions in a 0.1 M HCl coagulation bath.

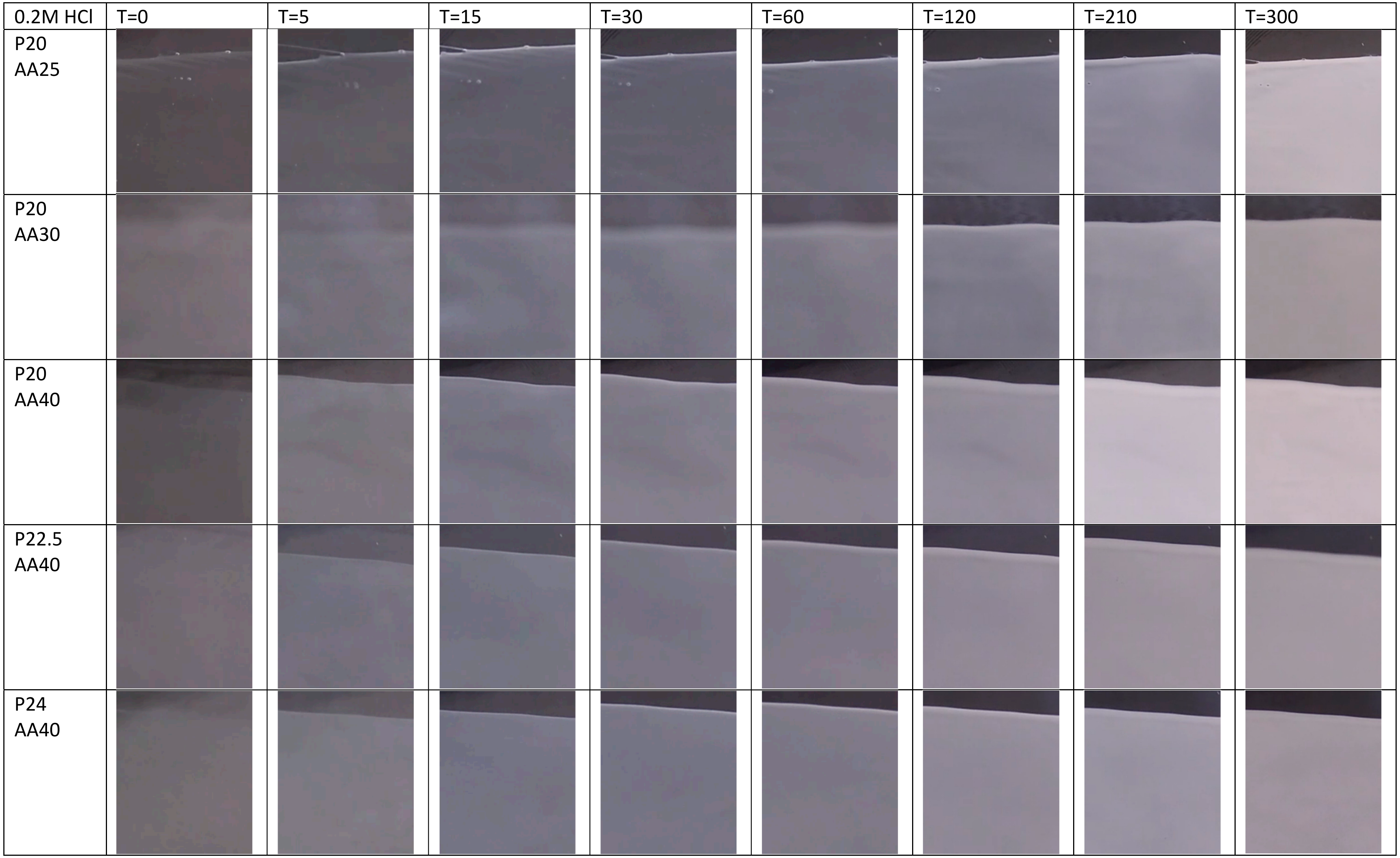

Figure S8. Frames taken at different times from movies of the precipitation of different polymer solutions in a 0.2 M HCl coagulation bath.

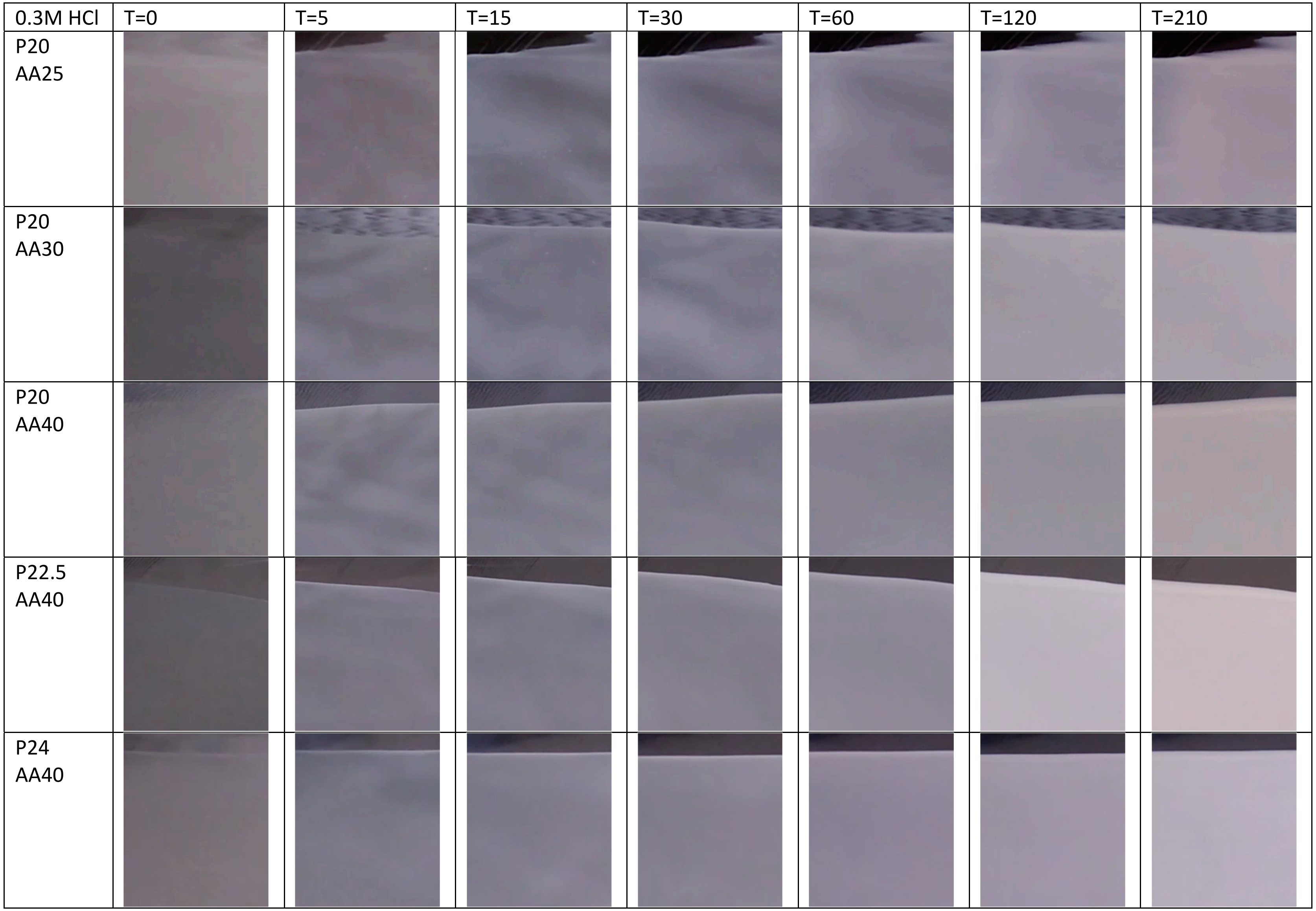

Figure S9. Frames taken at different times from movies of the precipitation of different polymer solutions in a 0.3 M HCl coagulation bath.

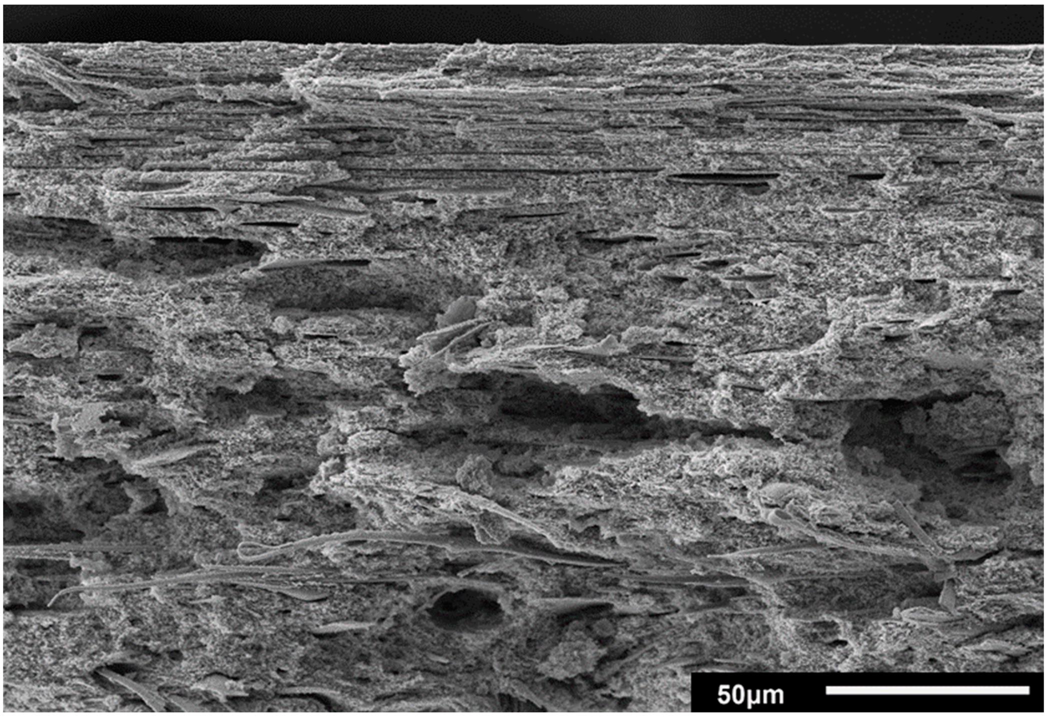

Figure S10. SEM image of the cross section of a membrane prepared with a PSaMA 20% w/v acetic acid 30% v/v solution in a coagulation bath with 0.1 M HCl.

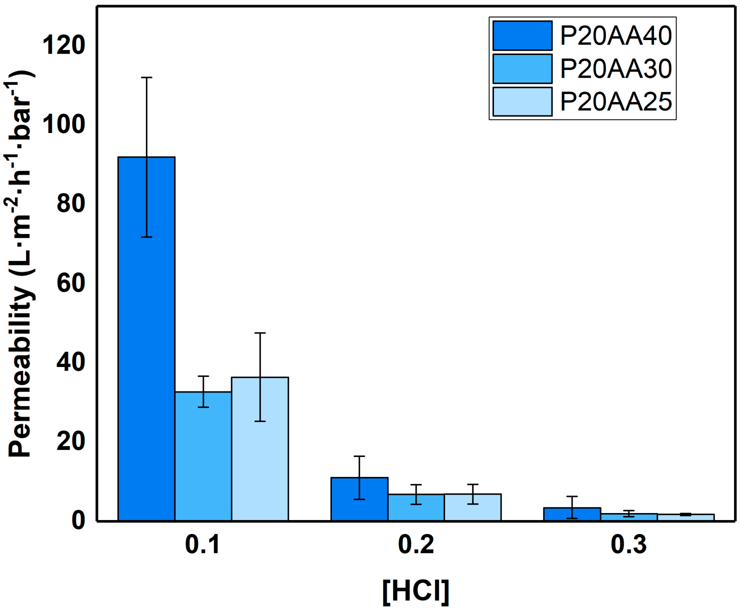

Figure S11. Pure water permeability of membranes prepared in a coagulation bath containing 0.1–0.3 M HCl using casting solutions containing 20% w/v PSaMA and either 40%, 30% or 25% v/v acetic acid. The error bars represent the sample standard deviation of at least three separate membranes.
